# Urban-rural disparities in heatwave effects on under-5 mortality in China, 2009–2019: a nationwide case-crossover study

**DOI:** 10.1016/j.lanwpc.2026.101922

**Published:** 2026-07-08

**Authors:** Chunhua He, Cheng He, Renjie Chen, Leni Kang, Yuxi Liu, Xue Yu, Jun Zhu, Yanping Wang, Haidong Kan

**Affiliations:** a National Office of Maternal and Child Health Surveillance of China, West China Second University Hospital, Sichuan University, Chengdu, China; b Key Laboratory of Birth Defects and Related Diseases of Women and Children, Sichuan University, Ministry of Education, Chengdu, China; c School of Public Health, Key Lab of Public Health Safety of the Ministry of Education and NHC Key Lab of Health Technology Assessment, Fudan University, Shanghai, China; d Children's Hospital of Fudan University, National Center for Children's Health, Shanghai, China

**Keywords:** Under-5 mortality, Heatwave, Climate change

## Abstract

**Background:**

Children under five years are physiologically susceptible to heat stress and significantly more affected by their surrounding environmental conditions, yet urban-rural differences in their vulnerability remain poorly understood, particularly regarding different heatwave timing patterns. We aimed to investigate whether and how the impacts of daytime, nighttime, and compound heatwaves on under-5 mortality differ between urban and rural areas of China.

**Methods:**

Using a nationwide case-crossover design, we analyzed 61,464 under-5 mortality cases across China from 2009 to 2019. High-resolution (1-km) temperature data were matched to each case location. We defined daytime, nighttime, and compound heatwaves as ≥2 consecutive days with maximum, minimum, or both temperatures exceeding the local 90th percentile, respectively. We employed a time-stratified case-crossover study design with conditional logistic regression and distributed lag models, adjusting for daily mean temperature, to estimate the cumulative effects of different heatwave events over 0–7 days. We examined eight principal causes of under-5 mortality, including preterm birth/low birth weight, birth asphyxia, pneumonia, diarrhea, leukemia, meningitis/encephalitis, and congenital cardiac defects. Stratified analyses were conducted by urban-rural residence, cause of death, age (neonatal: <28 days; post-neonatal: 28–364 days; early childhood: 1–4 years), sex, warm-season mean temperature, and temperature variability (standard deviation of daily mean temperatures from May to October), to identify vulnerable subgroups and potential effect modifiers.

**Findings:**

We analyzed 61,464 under-5 deaths (13,727 urban; 47,737 rural) across China from 2009 to 2019, with neonatal deaths (<28 days) constituting the largest age group in both urban (57.0%) and rural areas (53.8%). Nighttime and compound heatwaves were significantly statistically associated with increased under-5 mortality risk in rural areas (OR = 1.10, 95% CI: 1.03–1.17 and OR = 1.09, 95% CI: 1.03–1.16, respectively) but not in urban areas (OR = 0.96, 95% CI: 0.86–1.06 and OR = 0.95, 95% CI: 0.89–1.02, respectively), with significant urban-rural differences (p < 0.05). Rural vulnerability was particularly pronounced for child deaths related to preterm birth or low birth weight during both nighttime (OR = 1.18, 95% CI: 1.02–1.44) and compound heatwaves (OR = 1.21, 95% CI: 1.01–1.40). Post-neonatal infants and children in regions with lower mean temperature or temperature variability showed greater urban-rural disparities.

**Interpretation:**

Rural children in China are exposed to higher risks of heat-related mortality than their urban peers, especially during nighttime and compound heatwaves. Effective heat adaptation strategies should prioritize rural vulnerabilities and the threats posed by elevated nighttime temperatures in the context of a warming climate.

**Funding:**

10.13039/501100001809National Natural Science Foundation of China (82430105) and Shanghai Municipal Science and Technology Major Project (2023SHZDZX02).

**Research in context**

**Evidence before this study**

We searched PubMed and Web of Science up to January 2026 using the terms “heatwave”, “extreme heat”, “under-5 mortality”, “child mortality”, “urban-rural”, “daytime heatwave”, “nighttime heatwave”, and “compound heatwave”. Heatwaves pose a serious threat to vulnerable groups, including children under five. While past studies have linked extreme heat to higher mortality in many regions, few have examined how different types of heatwaves—daytime, nighttime, and compound—affect child mortality differently across urban and rural settings. Research to date has largely centered on urban populations, often overlooking rural communities that may be more at risk due to weaker infrastructure, limited healthcare access, and lower capacity to adapt.

**Added value of this study**

This study provides to the best of our knowledge the first comprehensive assessment of how different types of heatwaves affect under-5 mortality across urban and rural areas, using a large national dataset covering mortality cases from 2009 to 2019 in mainland China. By distinguishing between daytime, nighttime, and compound heatwaves, we identified significant rural-urban disparities in heat-related mortality risk. Our findings revealed that rural children are particularly vulnerable to nighttime and compound heatwaves, with specific cause-of-death patterns including pneumonia, diarrhea, and birth asphyxia showing elevated risk. We also demonstrated that these rural-urban differences persist after controlling for air pollution and are robust across different heatwave definitions.

**Implications of all the available evidence**

The findings highlight the need to tailor heat adaptation policies to account for rural-specific vulnerabilities, especially the risks associated with nighttime and compound heatwaves. Public health interventions should strengthen rural health systems and infrastructure to reduce the unequal burden of extreme heat on young children.

## Introduction

Climate change is driving an increase in the frequency, intensity, and duration of heatwaves globally,^1^ posing significant risks to human health.^2^ Children under five years of age represent a particularly vulnerable population due to their physiological characteristics and developing thermoregulatory systems.^3^ Compared to adults, young children have a higher surface area-to-volume ratio, limited ability to increase cardiac output, and less efficient sweating mechanisms relative to their body mass.^4^ These physiological constraints reduce their capacity to dissipate heat through evaporative and cardiovascular adjustments, making them more susceptible to rapid core temperature rises during extreme heat events.^5^ Additionally, children depend entirely on caregivers for appropriate protective actions, further increasing their vulnerability when heat adaptation resources or awareness are limited.^6^ Young children's limited ability to communicate their experience of heat-related physiological distress or discomfort further compounds this vulnerability, as caregivers may not recognize early signs of heat stress.

While heatwaves have traditionally been defined based on exceedances of daily mean or maximum temperatures,^7^^,^^8^ growing evidence suggests that the timing and diurnal pattern of extreme heat may have additional implications for health outcomes.^9^^,^^10^ Daytime heatwaves primarily impact outdoor activities and daytime exposures, while nighttime heatwaves disrupt physiological recovery and sleep quality,^11^ potentially leading to different pathophysiological responses.^12^ Compound heatwaves, characterized by concurrent extreme daytime and nighttime temperatures, may be associated with the greatest risk by combining acute daytime heat stress with impaired nighttime recovery.^13^ These distinctions are particularly important when considering urban-rural disparities in heat vulnerability, as the urban heat island effect typically results in higher ambient temperatures in cities compared to surrounding rural areas, particularly at night.^14^ However, this geographic temperature differential may be counterbalanced by disparities in adaptive resources. Rural areas often face challenges, including more limited healthcare access, fewer cooling resources, reduced electricity reliability, inadequate housing infrastructure, and lower socioeconomic status.^15^ These factors may render rural populations, especially vulnerable groups like children, more susceptible to heat-related health impacts despite potentially lower absolute temperatures. However, most heat health research has focused on urban populations or treated the entire city as a single entity, with relatively little attention to rural vulnerability or urban-rural comparisons.^16^ China presents an important context for examining this heat adaptation, as the whole country has experienced rapid urbanization and economic development in recent decades, creating pronounced socioeconomic gradients between urban and rural areas.^17^^,^^18^ Despite significant improvements in rural living standards, substantial differences persist in healthcare resources (with rural areas having approximately half the number of physicians per capita compared to urban areas^19^), housing quality, and cooling infrastructure.^19^ These disparities may be particularly consequential for heat vulnerability as China faces increasing heatwave frequency and intensity.^20^

Despite the significance of this issue, several critical knowledge gaps remain. First, few studies have distinguished between the health impacts of different heatwave types (daytime, nighttime, and compound),^13^^,^^21^ particularly among vulnerable populations such as children. Second, the mechanisms underlying potential urban-rural differences in heat vulnerability remain poorly understood, including how these might vary across different causes of death and regional climate characteristics. Addressing these knowledge gaps is essential for developing targeted heat adaptation strategies and health protection measures that account for the unique challenges faced by urban and rural communities in a rapidly warming climate.

In this study, we aimed to investigate whether and how the impacts of different heatwave types on under-5 mortality differ between urban and rural areas of China. Specifically, this study addresses the following research questions: (1) Do daytime, nighttime, and compound heatwaves have differential impacts on under-5 mortality between urban and rural areas? (2) Which causes of death and age groups show the greatest urban-rural disparities? (3) How do regional temperature characteristics modify these disparities?

We hypothesized that rural children would demonstrate greater heat vulnerability due to limited cooling resources and healthcare access, with nighttime and compound heatwaves posing particularly elevated risks. Using a nationwide case-crossover design spanning 2009–2019, we analyzed over 61,000 under-5 mortality cases to compare the effects of daytime, nighttime, and compound heatwaves across urban and rural settings. By distinguishing between different heatwave timing patterns and examining their differential impacts across the urban-rural divide, this study aims to provide novel insights into heat vulnerability that can inform more targeted adaptation strategies, as well as important implications for policy development in China and other countries experiencing rapid urbanization alongside increasing climate change impacts.

## Methods

### Mortality data for children under five years old

The mortality data for children under five years were obtained from the National Maternal and Child Health Surveillance System (MCHSS) spanning 2009 to 2019. Comprehensive descriptions of this surveillance system have been documented elsewhere.^22^^,^^23^ Specifically, MCHSS functions as a nationwide sample registration system collecting vital statistics on maternal and child death data. Currently, the surveillance network encompasses 334 monitoring sites distributed across China, comprising 124 urban sites and 210 rural sites. The classification of urban and rural areas followed the Chinese National Bureau of Statistics' urban-rural division regulations, where urban areas included municipal districts with high non-agricultural population density (≥1500 people/km2) while rural areas encompassed townships, villages, and county regions not incorporated into urban built-up areas. This system implements a methodological approach involving multi-stage, stratified, and cluster sampling techniques to ensure representativeness at both national and regional scales. All cases were restricted to deaths occurring in children below five years of age with permanent residence within the surveillance sites. Essential demographic and temporal information—including birth date, age, sex, home address, and time of death—was initially documented by village physicians or community practitioners, followed by systematic recording by community health personnel. Mortality causes were ascertained through hospital death certificates or medical certification/inference documents, and subsequently classified according to the International Classification of Diseases, Tenth Revision (ICD-10).^24^ Drawing upon our previous study,^23^ the current study focused on eight principal categories of non-accidental under-5 mortality: preterm birth/low birth weight complications (ICD-10: P07), birth asphyxia (P21), pneumonia (J12–J18), septicemia (A40–A41), diarrheal disease (A00–A09), leukemia (C91–C95), meningitis/encephalitis (G00–G09), and congenital cardiac defects (Q20–Q28). These categories encompass conditions with established or plausible heat-related pathways, including direct effects on respiratory and gastrointestinal infections,^25^^,^^26^ and indirect effects through maternal heat stress affecting uteroplacental function and birth outcomes.^27^^,^^28^ We included all major causes to provide a comprehensive mortality assessment.

Information on race or ethnicity was not collected in the surveillance system, as the study population is relatively homogeneous and such data are not routinely recorded in China's national mortality surveillance.

### Heatwave exposure assessment

Temperature data—including daily mean, maximum, and minimum temperatures—were derived from a high-resolution temperature dataset with 1 km spatial granularity covering mainland China from 2003 to 2022.^29^ This dataset integrates multiple data sources: meteorological station recordings, global seamless daily surface temperature measurements, and ancillary data from authoritative climate models. The comprehensive dataset was developed using a Four-Dimensional Spatio-Temporal Deep Forest model (4D-STDF) model with annual training and calibration procedures, yielding daily temperature measurements across China at 1 km resolution. Validation analysis demonstrated the dataset's accuracy and reliability, with root mean square errors of 1.49 °C for mean temperature, 1.53 °C for maximum temperature, and 1.18 °C for minimum temperature. This temperature dataset has been extensively utilized in previous temperature-related impact investigations. We geocoded residential addresses for each mortality case and matched the resulting geographical coordinates to the nearest 1-km grid cell, extracting time series data for daily mean, maximum, and minimum temperatures throughout the study period. Additionally, based on established methodologies from a previous study,^23^ we obtained daily measurements of air pollutants (PM_2.5_, NO_2_, SO_2_, O_3_) from the closest air quality monitoring stations within the National Air Quality Monitoring System.^30^

To investigate differential impacts of heatwaves occurring at various times of day, and building upon our recent research,^13^ we implemented three distinct heatwave definitions. Daytime heatwaves were characterized as periods when maximum daily temperatures exceeded the local 90th percentile threshold for at least two consecutive days. Nighttime heatwaves were defined as instances when minimum daily temperatures surpassed the local 90th percentile threshold for a minimum of two consecutive days. Compound heatwaves were identified when both daytime and nighttime criteria were simultaneously satisfied. We conducted individualized heatwave identification for each case location based on local temperature distributions throughout the study period from 2009 to 2019, thereby establishing specific heatwave exposure profiles for each geographical location.

### Statistical analysis

The mortality impact of heatwave exposure among children under five years was examined using a time-stratified case-crossover study design.^31^^,^^32^ Under this methodological setting, we designated the death date as the case day for each mortality event. Corresponding control days were identified as all other days falling within the identical month and year as the case day, while also matching the specific day of the week (both before and after the case day). This bidirectional, time-symmetric selection eliminates potential bias from within-month temporal trends. This method also allows each case to serve as his/her own control, thus, individual level covariates (such as sex, age, and other socioeconomic status) that do not vary in the short term could be controlled as they remain constant for the same person. This strategy also can effectively mitigate potential confounding influences related to weekly patterns, seasonal variations, and longitudinal temporal trends.

We employed a conditional logistic regression as the main analysis model. In this main model, mortality events are treated as the dependent variable, and the heatwave occurrence indicator as a binary variable serves as the independent variable. To capture the temporal lag effects of heatwave events, we implemented a distributed lag model (DLM).^33^ A maximum lag of 7 days (0–6 d) was selected based on our previous studies and the case-crossover study design.^12^ The lag-response function was built using a natural cubic B-spline with 4 degrees of freedom (df) and two internal knots placed at equally spaced values in the log scale.^8^^,^^34^ In the main model, we also adjusted for daily mean temperature using a distributed lag non-linear model (DLNM). Specifically, we introduced a cross-basis function of daily mean temperature, which includes a quadratic B-spline with two internal knots placed at the 50th and 90th centiles of daily temperature distributions, as done in previous studies.^12^^,^^34^ The lag response curve was set with a natural cubic B-spline with an intercept and three internal knots placed at equally spaced values in the log scale, with the same maximum lag of up to 7 days.^12^ Following current methodological standards, we adjusted for daily mean temperature to identify the independent effect of heatwave events^33^^,^^35^

To evaluate differential impacts of heatwaves between urban and rural locations, we performed separate analyses for all three heatwave types across urban and rural mortality cases. We also disaggregated our analysis by examining each of the eight major causes of death individually. To determine whether differences between urban and rural effect estimates were statistically significant, we implemented the multivariate Wald test, with p-values below 0.05 considered statistically significant.^12^^,^^36^ For more details on this part of the method, please refer to the Supplementary Methods Section in the Supplementary Material.

Additionally, we conducted stratified analyses based on developmental periods (neonatal: <28 days; post-neonatal: 28–364 days; early childhood: 1–4 years) and child sex (male and female). We also stratified by baseline temperature characteristics of the locations, including warm season mean temperature levels and temperature variability (measured as the standard deviation of daily mean temperatures during warm months from May to October from 2009 to 2019), acknowledging that heatwaves predominantly occur during warmer months and that local climatic baseline conditions can influence the adaptive capacity of residents in a given region to temperature extremes.

To validate the robustness of our findings, we performed multiple sensitivity analyses. First, we examined alternative heatwave definitions by varying intensity thresholds (using the 95th percentile) and minimum duration requirements (extending to 3 consecutive days). Second, we augmented our main model with natural splines for four distinct air pollutants to assess the potential confounding effects of air pollution. Third, considering the substantial difference in case numbers between urban and rural areas in our study, we employed a spatially and cause-matched balanced samples approach to verify that our main findings remained significant under conditions of equal urban and rural case distribution. This method ensured that each urban case was matched with a rural case of the same cause of death and closest spatial proximity, thereby eliminating potential bias from sample size imbalance. Finally, we tested key model parameters related to the heatwave exposure–response relationship. For more details on these sensitivity tests, please refer to the supplementary materials.

All statistical procedures were executed using R software. Results are presented as odds ratios with corresponding 95% confidence intervals (95% CI), representing the risk of mortality during heatwave periods compared to non-heatwave days.

Ethical approval was not required, as this study was a secondary analysis of de-identified, routinely collected surveillance data.

### Role of the funding source

The funders had no role in study design, data collection and analysis, decision to publish, or preparation of this study.

## Results

**Fig. 1:**
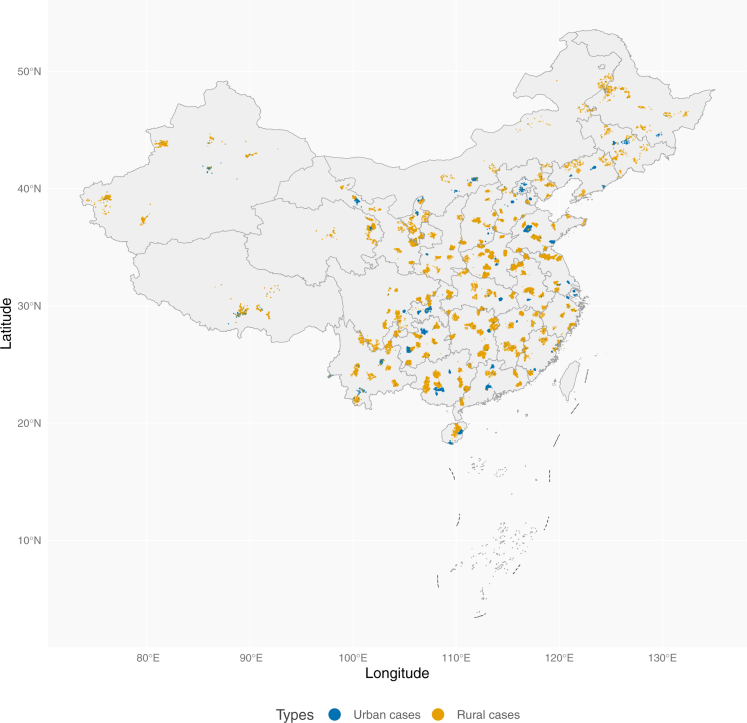
**Spatial distribution of under-5 mortality cases included in the study across urban (blue) and rural (red) surveillance sites in China, 2009–2019**.

Table 1 summarizes the basic characteristics of the study population. During the study period from January 2009 to December 2019, we identified a total of 61,464 under-5 mortality cases from all causes (Fig. 1 and Table 1). Rural areas accounted for 47,737 total death cases, and urban areas accounted for 13,727 death cases. Both regions primarily reported preterm birth/low birth weight, birth asphyxia, congenital heart disease, and pneumonia as the main causes, though with varying proportions. By age stages, neonatal deaths (<28 days) constituted the largest proportion in both regions, accounting for 57.0% (7821/13,727) in urban areas and 53.8% (25,670/47,737) in rural areas. The proportion of deaths in children aged 1–4 years was comparable between urban areas (2525, 18.4%) and rural areas (8,638, 18.1%). Regarding sex distribution, the male-to-female ratio was similar in urban (1.25) and rural areas (1.28). For temperature characteristics, Table S1 shows comparable warm-season (May–October) mean temperatures between urban (22.7 °C [19.3 °C, 24.9 °C]) and rural areas (22.4 °C [19.5 °C, 23.9 °C]) where all under-5 mortality cases permanently resided. Annual heatwave patterns were similar, with both regions experiencing approximately 31 days of daytime and nighttime heatwaves. Urban areas had slightly more compound heatwave days (17 d [14 d, 19 d]) than rural areas (16 d [12 d, 19 d]). Temperature variability stratification indicated that the proportion of rural death cases in high temperature variability regions (>3.46 °C) was lower (33.0%) than in urban areas (41.0%). Additionally, Figures S1 and S2 demonstrate that the spatial distribution of temperature standard deviation and mean temperature were generally consistent, with lower temperature areas experiencing greater fluctuations. However, some regions such as the Central Plains showed both relatively high mean temperatures and higher standard deviations.

**Table 1:** Distribution of characteristics among included under-5 mortality cases in urban and rural areas of China.

	Urban	Rural
Total under-5 deaths		
Total	13,727	47,737
Neonatal mortality (<28 days)	7821 (57.0%)	25,670 (53.8%)
Post-neonatal morality (28–364 days)	3381 (24.6%)	13,429 (28.1%)
Mortality from 1 year to 4 years	2525 (18.4%)	8638 (18.1%)
Different causes		
Preterm birth/Low birth weight	2799 (20.4%)	8649 (18.1%)
Birth asphyxia	1937 (14.1%)	7008 (14.7%)
Pneumonia	649 (4.7%)	4187 (8.8%)
Septicemia	558 (4.1%)	1283 (2.7%)
Diarrhea	194 (1.4%)	1648 (3.5%)
Leukemia	280 (2.0%)	806 (1.7%)
Meningitis/Encephalitis	186 (1.4%)	883 (1.8%)
Congenital heart disease	894 (6.5%)	3821 (8.0%)
Other conditions	6230 (45.4%)	19,452 (40.7%)
Sex		
Male	7616 (55.5%)	26,742 (56.0%)
Female	6088 (44.4%)	20,927 (43.8%)
NA^a^	23 (0.1%)	68 (0.1%)
Mean temperature during the warm months		
<21.22 °C	5563 (40.5%)	19,412 (40.7%)
21.22–23.66 °C	3154 (23.0%)	15,189 (31.8%)
>23.66 °C	5010 (36.5%)	13,136 (27.5%)
Warm-season temperature variability (Standard deviation)		
<2.98 °C	4556 (33.2%)	16,455 (34.5%)
2.98–3.46 °C	3537 (25.8%)	15,506 (32.5%)
>3.46 °C	5634 (41.0%)	15,776 (33.0%)

a NA, not available.

**Fig. 2:**
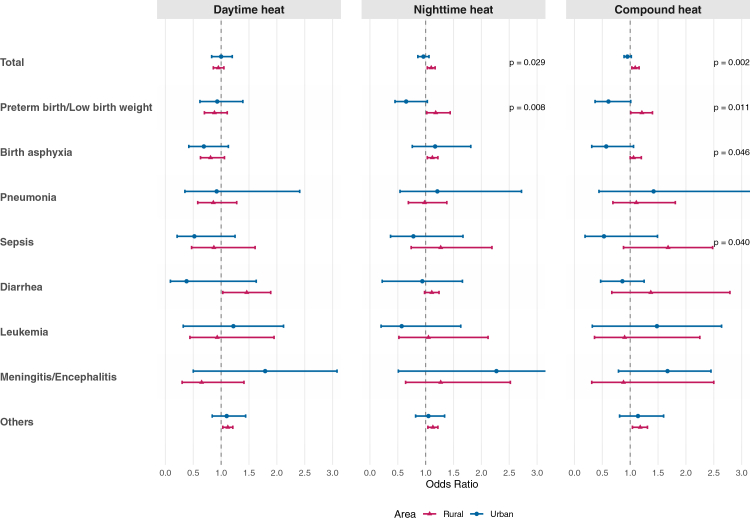
**Comparative effects of daytime heatwaves, nighttime heatwaves, and compound heatwaves on cause-specific under-5 mortality in urban and rural areas.** The odds ratios represent cumulative effects over lag 0–7 days after controlling for mean temperature effects. The dots represent point estimates, and the lines indicate 95% confidence intervals. Daytime heatwaves were defined as days when maximum temperature exceeded the 90th percentile for two consecutive days, nighttime heatwaves as days when minimum temperature exceeded the 90th percentile for two consecutive days, and compound heatwaves as days meeting both criteria simultaneously. p-values indicate statistically significant differences between urban and rural results (p < 0.05).

Fig. 2 illustrates the comparative effects of different heatwave types on under-5 mortality in urban and rural areas. While daytime heatwaves showed no significant effect on under-5 mortality in either urban (OR = 1.00, 95% CI: 0.83–1.20) or rural areas (OR = 0.95, 95% CI: 0.86–1.05), both nighttime and compound heatwaves exhibited significant impacts exclusively in rural settings. For nighttime heatwaves, the cumulative risk over lag 0–7 days was significantly elevated in rural areas (OR = 1.10, 95% CI: 1.03–1.17) but not in urban areas (OR = 0.96, 95% CI: 0.86–1.06). Similarly, compound heatwaves were associated with increased mortality risk in rural areas (OR = 1.09, 95% CI: 1.03–1.16) while showing no significant effect in urban areas (OR = 0.95, 95% CI: 0.89–1.02). The urban-rural differences for both nighttime and compound heatwave effects were statistically significant (p < 0.05). Lag patterns (Figures S3–S5) revealed that the excess mortality risk in rural areas primarily manifested within 0–3 days after heatwave exposure, with the peak effect observed around lag day 1–2, followed by a gradual decline.

Among specific causes of death, preterm birth/low birth weight showed pronounced urban-rural disparities. While rural neonates (<28 days) exhibited significantly associated with elevated risk during both nighttime heatwaves (OR = 1.18, 95% CI: 1.02–1.44) and compound heatwaves (OR = 1.21, 95% CI: 1.01–1.40), urban neonates demonstrated no significant increased risk (nighttime: OR = 0.65, 95% CI: 0.45–1.03; compound: OR = 0.61, 95% CI: 0.37–1.01). These urban-rural differences were highly significant (p = 0.0076 for nighttime; p = 0.0108 for compound heatwaves). Birth asphyxia also showed significant urban-rural differences for compound heatwaves (p = 0.046), with rural infants exhibiting borderline increased risk (OR = 1.06, 95% CI: 1.00–1.20) compared to urban infants (OR = 0.57, 95% CI: 0.31–1.06). For diarrhea, rural children showed increased risk during daytime heatwaves (OR = 1.46, 95% CI: 1.03–1.89), while urban children showed no such increase, though this urban-rural difference did not reach statistical significance (p = 0.068).

**Fig. 3:**
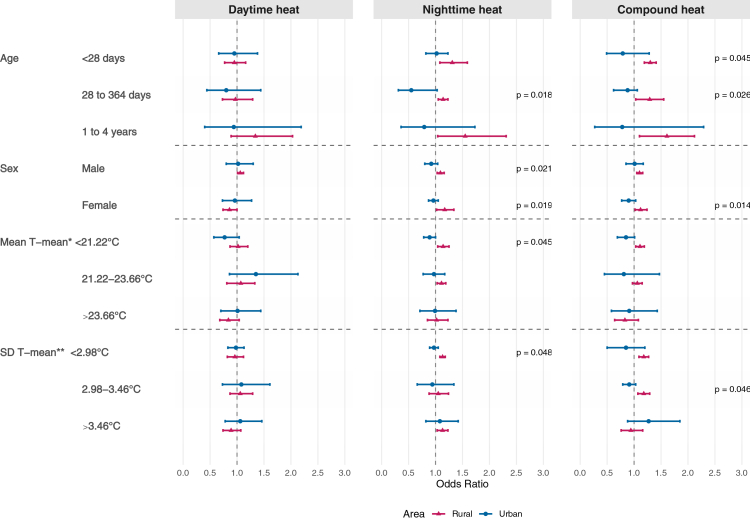
**Stratified effects of different heatwave types on under-5 mortality in urban and rural areas by age, sex, and temperature characteristics.** p-values indicate statistically significant differences between urban and rural results (p < 0.05).

As shown in Fig. 3, age-stratified analysis revealed that the rural-urban disparity was most pronounced among post-neonatal infants (28–364 days) and neonates (<28 days). In this age group, rural infants exhibited higher risk during nighttime heatwaves and compound heatwaves compared to their urban counterparts. Children aged 1–4 years in rural areas also demonstrated vulnerability to nighttime heatwaves, though the urban-rural difference was less substantial. In regions with lower warm month mean temperatures (<21.22 °C), rural children showed significantly increased risk during both nighttime heatwaves and compound heatwaves. The urban-rural difference was statistically significant (p < 0.05). Similarly, in regions with low temperature variability (standard deviation < 2.98 °C), rural children exhibited significantly greater vulnerability to nighttime heatwaves compared to urban children in areas with similar temperature variability.

Table S2 presents our comprehensive sensitivity analyses, which affirmed the robustness of the main findings. When adjusting for key air pollutants (PM_2.5_, O_3_, and NO_2_), the observed urban-rural disparity in heatwave vulnerability remained consistent. Changing the degrees of freedom for the lag-response function from 3 to 5 also yielded consistent results (Table S2). We further tested alternative heatwave definitions with more stringent criteria. Using a higher temperature threshold (95th percentile for two consecutive days), as shown Table S3, the results remained consistent. Table S4 demonstrates that after implementing the case-balanced analysis with equal urban and rural sample sizes (Figure S6), our main findings remained largely unchanged, with rural areas still showing significantly elevated mortality risks during nighttime (OR = 1.19, 95% CI: 1.04–1.38) and compound heatwaves (OR = 1.11, 95% CI: 1.02–1.22).

## Discussion

In this nationwide study spanning 2009–2019, we examined the differential impacts of three different timing types of heatwaves on under-5 mortality across urban and rural areas of China. Using a case-crossover design, we found that both nighttime and compound heatwaves were significantly associated with increased mortality risk among rural children, while urban children showed less vulnerability. This was particularly evident for preterm birth/low birth weight and birth asphyxia cases occurring in rural areas. Age-stratified analyses revealed similar patterns for both post-neonatal infants and neonatal deaths. In addition, rural children in regions with lower baseline temperatures and weaker temperature variability faced particularly elevated risks during heatwaves. Our approach of distinguishing between daytime, nighttime, and compound heatwaves provides novel insights beyond previous research, revealing that the children's inability to recover during cooler nighttime periods may be more detrimental than daytime heat stress alone, particularly in resource-limited rural settings.

Previous research on heat-related mortality has predominantly focused on urban populations,^16^ often highlighting urban heat island effect as a exacerbating factor.^37^ Studies from some high-income countries have established significant heat-related mortality risks in urban areas, or treating the entire city as a single entity, but rarely explored urban-rural differences.^38^ Our finding that rural children in China face greater heat vulnerability represents an important departure from this urban-centric perspective. This pattern aligns with limited evidence from other developing regions, which suggest that limited infrastructure and healthcare access may override urban heat island advantages.^39^ Additionally, our temporal differentiation between heatwave types reveals the critical importance of nighttime temperatures—an aspect overlooked in many previous studies that relied on daily mean or maximum temperatures to define the extreme heat events.^8^^,^^34^ While recent work has begun to highlight the importance of minimum temperatures or compound heat events,^12^^,^^13^ our findings on the specific vulnerability of rural infants to nighttime heatwaves provide unprecedented insights into this relationship. Furthermore, our cause-specific analysis identifies particular pathways of vulnerability (e.g., preterm birth complications) that extend beyond the cardiovascular and respiratory outcomes typically emphasized in adult-focused heat mortality research.^21^ Moreover, our examination of effect modification by regional temperature characteristics adds a novel dimension to understanding heat vulnerability—showing that areas with lower temperature variability and those with relatively lower baseline temperatures may face significant risks, consist to assumptions that populations in cooler regions are more vulnerable due to climate-driven warming.^40^ These findings contribute to a growing body of evidence suggesting that heat adaptation strategies must be context-specific and population-sensitive rather than universally applied.^41^

The observed urban-rural disparity in heat vulnerability likely explained by multiple interconnected mechanisms. Physiologically, infants and young children are inherently vulnerable to heat stress due to their higher surface area-to-volume ratio,^42^ limited thermoregulatory capacity, and greater metabolic heat production relative to adults.^43^ Children have less developed sweat glands and relatively higher body water content, making their thermoregulation less efficient in high-temperature environments.^44^ This vulnerability is potentially modified in rural settings through significant environmental and socioeconomic disparities. The absence of significant heatwave effects in urban areas does not necessarily indicate that urban children are immune to heat stress, but rather suggests that urban advantages in adaptive resources may effectively mitigate heat-related mortality risks. Rural dwellings in China typically offer less protection against extreme temperatures, with poorer insulation, fewer cooling appliances, and greater reliance on natural ventilation, especially in the northern part of China. Data shows that as of 2023, rural households possessed only 105 air conditioners per 100 households compared to 163 in urban areas.^45^ The sensitivity to nighttime heatwaves we observed suggests that sustained thermal strain without adequate recovery periods may be especially detrimental. Healthcare access disparities further compound these effects, with approximately 6.88 hospital beds per 1000 people in urban areas, which is 2.2 times higher than in rural areas in 2015.^46^ For conditions like preterm birth complications and birth asphyxia, delayed medical intervention during heat-related deterioration could be particularly consequential. Nutritional status may also play a role, data shows that stunting rates among rural children under five (12.1%) were nearly triple those of urban children (3.4%),^47^ potentially reducing their physiological resilience to heat stress. Socioeconomic factors create additional disadvantages, with rural residents' per capita disposable income (20,133 RMB in 2022) being less than half that of urban residents (49,283 RMB),^48^ limiting investment in -cooling equipment and its usage. Caregiving patterns also differ significantly. China's rural areas have approximately 61 million “left-behind children” primarily cared for by grandparents,^49^ who may be more reluctant to use electrical cooling devices due to cost concerns and may have different levels of health literacy regarding heat risks and infant care practices, including recognition of heat-related warning signs. These multifaceted mechanisms likely interact synergistically, creating a compound disadvantage for rural children during heatwaves, particularly when temperatures remain elevated throughout the night, preventing physiological recovery.

Our findings have several important policy implications for child health protection in the context of climate change. First, heat action plans and early warning systems should be designed to address the specific vulnerabilities of rural communities, with particular attention to nighttime temperature and compound heat events that our study identifies as critical for child health. These systems should utilize accessible communication channels appropriate for rural settings and provide location-specific guidance. Second, healthcare resources for managing heat-sensitive conditions like birth complications should be strategically strengthened in rural areas, such as improved emergency referral systems during extreme heat events. Third, economic support mechanisms could include subsidized cooling equipment for vulnerable households with young children and temporary electricity subsidies during prolonged heatwaves. Fourth, our results highlight the need for enhanced vigilance in regions with relatively lower baseline temperatures and temperature variability, where populations may have less developed physiological and infrastructural adaptations to heat. Finally, community-based interventions should engage village doctors, schools, and local leaders in identifying at-risk children and establishing community cooling centers that can serve as emergency refuges during extreme heat events. These measures should be integrated into broader rural development and healthcare equity initiatives to address this critical environmental health disparity. Future studies using complete population-based mortality registration data would be valuable to quantify the absolute burden and population attributable fraction of heat-related under-5 mortality, which would further inform resource allocation and policy prioritization.

Limitations of this study should be acknowledged. First, while our case-crossover design controlled for personal confounders and our stratified analyses provided insights into vulnerability patterns, we were unable to directly measure indoor temperatures, which may lead to some exposure misclassification bias. Studies integrating personal temperature monitoring in rural households would provide more accurate exposure assessment. Furthermore, we used air temperature to define heatwaves, while composite thermal indices (e.g., heat index, WBGT) that incorporate humidity may better reflect physiological heat stress.^50^ Second, though we adjusted for major air pollutants and considered temperature variability, other environmental or socioeconomic factors could have influenced our results. In addition, we addressed several knowledge gaps identified in the urban-rural disparities. We could not directly measure household-level factors such as air conditioning availability, housing quality, caregiver health literacy, or healthcare utilization patterns that likely mediate the observed urban-rural differences. Future research should explore the mechanisms underlying the observed urban-rural disparity by incorporating household surveys on household-level factors. Additionally, this study only discussed potential mechanisms of action, but intervention studies testing the effectiveness of targeted cooling strategies, early warning systems, and community-based programs in rural areas are needed to translate these findings into actionable policies. Third, our surveillance system does not record the place of death, which limits our ability to determine whether mortality occurred at home, in healthcare facilities, or during transport. Future studies incorporating this information could help prioritize whether interventions should focus more on residential cooling infrastructure or on healthcare accessibility. Temperature exposure was assessed based on residential addresses, which may not fully capture daytime exposures for children attending childcare facilities. However, given the low childcare enrollment rates during the study period and the predominance of neonatal deaths, residential addresses likely represent the primary exposure setting for most cases. Finally, this study examined mortality rather than morbidity outcomes. Future research comparing heat-related morbidity-to-mortality ratios between urban and rural areas could help clarify whether the observed disparities stem primarily from differences in disease incidence or healthcare effectiveness. If morbidity rates are similar but mortality differs, this would indicate that timely medical intervention plays a critical protective role, thereby strengthening evidence for healthcare infrastructure improvements in rural settings.

In conclusion, our nationwide study reveals a strong urban-rural disparity in heat vulnerability among children under five in China, with rural children facing higher health risks during nighttime and compound heatwaves. This rural disadvantage was particularly pronounced for conditions related to birth complications, with neonatal and post-neonatal infants showing the higher vulnerability. The absence of significant effects during daytime heatwaves, coupled with the pronounced impact of nighttime heat, highlights the critical importance of thermal recovery periods and suggests that conventional heat warning systems focusing primarily on daytime maximum temperatures may inadequately protect vulnerable rural populations. Our findings underscore the need for targeted heat adaptation strategies addressing the specific challenges faced by rural communities, particularly as recent warming intensifies extreme heat events. Protecting children from heat-related mortality requires not only enhanced early warning systems and healthcare access but also addressing underlying socioeconomic and infrastructural disparities between urban and rural areas in China.

## Contributors

ChH, CH, and RC designed the study. ChH, LK, YL, and XY collected the data. ChH and CH conducted the statistical analyses. ChH, CH, RC, and HK verified the underlying data. ChH and CH drafted the manuscript. All authors contributed to data interpretation, critically revised the manuscript for important intellectual content, and approved the final version. JZ, YW, and HK supervised the study.

## Data sharing statement

The mortality data analyzed in this study were obtained from the National Maternal and Child Health Surveillance System (MCHSS) of China, which is managed by the National Office of Maternal and Child Health Surveillance, West China Second University Hospital, Sichuan University. Access to individual-level mortality data is restricted due to privacy and confidentiality regulations. Data supporting the findings of this study are only available from the corresponding authors upon reasonable request. The high-resolution temperature dataset used in this study is publicly available from https://doi.org/10.1038/s41597-024-03980-z. Statistical code for data analysis is available from the corresponding authors upon reasonable request.

## Editor note

Nationwide surveillance data from China show that nighttime and compound day-night heatwaves are linked to significantly higher death risk among rural under-5 children, with less significant risk among urban children, highlighting nighttime heat as an overlooked risk requiring rural-focused adaptations.

## Declaration of interests

All authors declare no competing interests related to this work.
