# Supplementary material for: Urban-rural disparities in heatwave effects on under-5 mortality in China, 2009–2019: a nationwide case-crossover study
Source: Lancet Reg Health West Pac. 2026 Jul 8;72:101922. doi: 10.1016/j.lanwpc.2026.101922 (PMC13380143; doi:10.1016/j.lanwpc.2026.101922)
Supplement: Supplementary file [file mmc1.docx]

**Supplementary Material**

**Methods for testing the statistical significance of the difference**

To test the statistical significance of the difference in odds ratios (ORs) between the two time periods over different subgroups, according to a related study^1^, we calculated the z score as:

$$z=\frac{(E_{1}-E_{2})}{\sqrt{{(SE_{1})}^{2}+{(SE_{2})}^{2}}}$$

Where $E_{1}$ and $E_{2}$ are the natural logarithms of the estimated OR, $SE_{1}$ and $SE_{2}$ are their respective standard errors calculated from their estimated 95% CIs.

**Sensitivity analysis**

As we mentioned in the main text, we conducted series of sensitivity analyses of our analysis from several aspects including:

1. Definition of heat waves

In addition to the definition used in the main analysis (90^th^ percentile 2 consecutive days), we used two alternative thresholds to define three different heat waves, including varying intensity thresholds (using the 95th percentile) and minimum duration requirements (extending to 3 consecutive days)

1. Confounder effect of air pollution

To identify whether there is a potential effect modification of air pollutants on the impact of different heat waves, we also added three four types of air pollutants, including PM_2.5_, O_3,_ and NO_2_, in the main model, separately.

1. Spatially and cause-matched balanced samples approach

To address this methodological concern and validate that our observed urban-rural disparities were not merely artifacts of sample size differences, we developed a spatially and cause-matched balanced samples approach. This approach included several steps, first, for each urban mortality case (13,727), we identified rural cases with identical causes of death to ensure comparability, then calculated the spatial distance between each urban case and all corresponding rural cases based on latitude and longitude coordinates. We then selected the geographically closest rural case with an identical cause of death for each urban case, creating one-to-one matched pairs while ensuring each rural case was used only once. This process yielded a balanced dataset with equal numbers of urban and rural cases, matched on both causes of death and spatial proximity. The spatial distribution of the final matched results is shown in Figure S6. After that, we repeated our main analytical procedures, applying the time-stratified case-crossover design with conditional logistic regression and distributed lag models to examine the differential impacts of daytime, nighttime, and compound heat waves on under-5 mortality between urban and rural areas.

1. Key parameters in the main model

As suggested by the related temperature effect study ^2^, some settings of the key parameters in our main model may affect the estimate results, such as the knots setting, so, we adjusted the knots for exposure-response accordingly and to see if our findings were still stable.

**Table S1 Comparison of warm-season temperature and annual heat wave day characteristics between urban and rural case locations, 2009-2019.** Data are presented as median [25th percentile, 75th percentile] of all locations. Warm season is defined as May to October of each year. Daytime heat waves are defined as days when the daily maximum temperature exceeded the local 90th percentile for two or more consecutive days. Nighttime heat waves are defined as days when the daily minimum temperature exceeded the local 90th percentile for two or more consecutive days. Compound heat waves are defined as days when both daytime and nighttime heat wave criteria were met simultaneously.

|  | Urban | Rural |
| --- | --- | --- |
| Mean temperature | 22.7 [19.3, 24.9] | 22.4 [19.5, 23.9] |
| Number of daytime heatwave days per year | 31 [29, 32] | 31 [29, 32] |
| Number of nighttime heatwave days per year | 31 [30, 32] | 31 [30, 32] |
| Number of compound heatwave days per year | 17 [14, 19] | 16 [12, 19] |

**Table S2 Sensitivity analysis of heat wave effects on under-5 mortality in urban and rural areas with adjustments for air pollutants and different model specifications.** Data are presented as odds ratios with 95% confidence intervals in brackets. Results show the cumulative effects over lag 0-6 days for different heat wave types (daytime, nighttime, and compound) in urban and rural areas. The unadjusted model controls for mean temperature, while subsequent models additionally adjust for different air pollutants (PM_2.5_, O_3_, and NO_2_) individually. The final model tests the robustness of results using a different degrees of freedom (df=5) for the lag-response function. All odds ratios represent the comparison between heat wave days and non-heat wave days.

|  |  | Daytime | Nighttime | Compound |
| --- | --- | --- | --- | --- |
| Unadjusted | Urban | 1.00 [0.83-1.20] | 0.96 [0.86-1.06] | 0.95 [0.89-1.02] |
|  | Rural | 0.95 [0.96-1.05] | 1.10 [1.03-1.17] | 1.09 [1.03-1.16] |
|  |  |  |  |  |
| Adjusting for PM_2.5_ | Urban | 1.02 [0.85-1.22] | 0.97 [0.87-1.07] | 0.96 [0.90-1.03] |
|  | Rural | 0.96 [0.88-1.06] | 1.12 [1.05-1.19] | 1.10 [1.04-1.17] |
|  |  |  |  |  |
| Adjusting for O_3_ | Urban | 1.01 [0.84-1.21] | 0.96 [0.86-1.06] | 0.96 [0.90-1.03] |
|  | Rural | 0.97 [0.89-1.07] | 1.11 [1.04-1.18] | 1.10 [1.04-1.17] |
|  |  |  |  |  |
| Adjusting for NO_2_ | Urban | 1.01 [0.84-1.21] | 0.96 [0.86-1.07] | 0.96 [0.90-1.03] |
|  | Rural | 0.98 [0.89-1.07] | 1.12 [1.05-1.19] | 1.11 [1.05-1.18] |
| Df for lag-response: |  |  |  |  |
| 5 | Urban | 1.03 [0.86-1.23] | 0.97 [0.87-1.08] | 0.97 [0.91-1.04] |
|  | Rural | 0.98 [0.90-1.08] | 1.13 [1.06-1.20] | 1.12 [1.06-1.19] |

**Table S3 Sensitivity analysis of heat wave effects on under-5 mortality using different heat wave definitions.** Data are presented as odds ratios with 95% confidence intervals in brackets. Results show the cumulative effects over lag 0-6 days for different heat wave types (daytime, nighttime, and compound) in urban and rural areas. The unadjusted model uses the baseline definition (90th percentile threshold for 2 consecutive days). Alternative definitions include: '90% 3d' (90th percentile threshold for 3 consecutive days) and '95% 2d' (95th percentile threshold for 2 consecutive days). All odds ratios represent the comparison between heat wave days and non-heat wave days.

|  |  | Daytime | Nighttime | Compound |
| --- | --- | --- | --- | --- |
| Unadjusted | Urban | 1.00 [0.83-1.20] | 0.96 [0.86-1.06] | 0.95 [0.89-1.02] |
|  | Rural | 0.95 [0.96-1.05] | 1.10 [1.03-1.17] | 1.09 [1.03-1.16] |
|  |  |  |  |  |
| 90% 3d | Urban | 1.13 [0.87-1.47] | 0.99 [0.87-1.07] | 0.98 [0.85-1.13] |
|  | Rural | 1.13 [0.97-1.31] | 1.13 [1.06-1.30] | 1.15 [1.07-1.24] |
|  |  |  |  |  |
| 95% 2d | Urban | 1.42 [0.98-1.86] | 1.00 [0.84-1.23] | 1.11 [0.86-1.36] |
|  | Rural | 1.23 [0.97-1.49] | 1.24 [1.14-1.34] | 1.42 [1.12-1.74] |

**Table S4 Comparison of heat wave effects on under-5 mortality between the main analysis and balanced sample analysis.** Data are presented as odds ratios with 95% confidence intervals in brackets. Results show the cumulative effects over lag 0-6 days for different heat wave types (daytime, nighttime, and compound) in urban and rural areas. The top panel shows results from the main analysis with all available cases (urban: n=13,727; rural: n=47,737), while the bottom panel displays results from the balanced sample analysis with equal case numbers (n=11,264 each) matched on cause of death and spatial proximity.

|  |  | Daytime | Nighttime | Compound |
| --- | --- | --- | --- | --- |
| Main results | Urban | 1.00 [0.83-1.20] | 0.96 [0.86-1.06] | 0.95 [0.89-1.02] |
|  | Rural | 0.95 [0.96-1.05] | 1.10 [1.03-1.17] | 1.09 [1.03-1.16] |
|  |  |  |  |  |
| Case balanced | Urban | 1.00 [0.84-1.20] | 0.96 [0.86-1.06] | 0.95 [0.89-1.02] |
|  | Rural | 0.88 [0.73-1.05] | 1.19 [1.04-1.38] | 1.11 [1.02-1.22] |


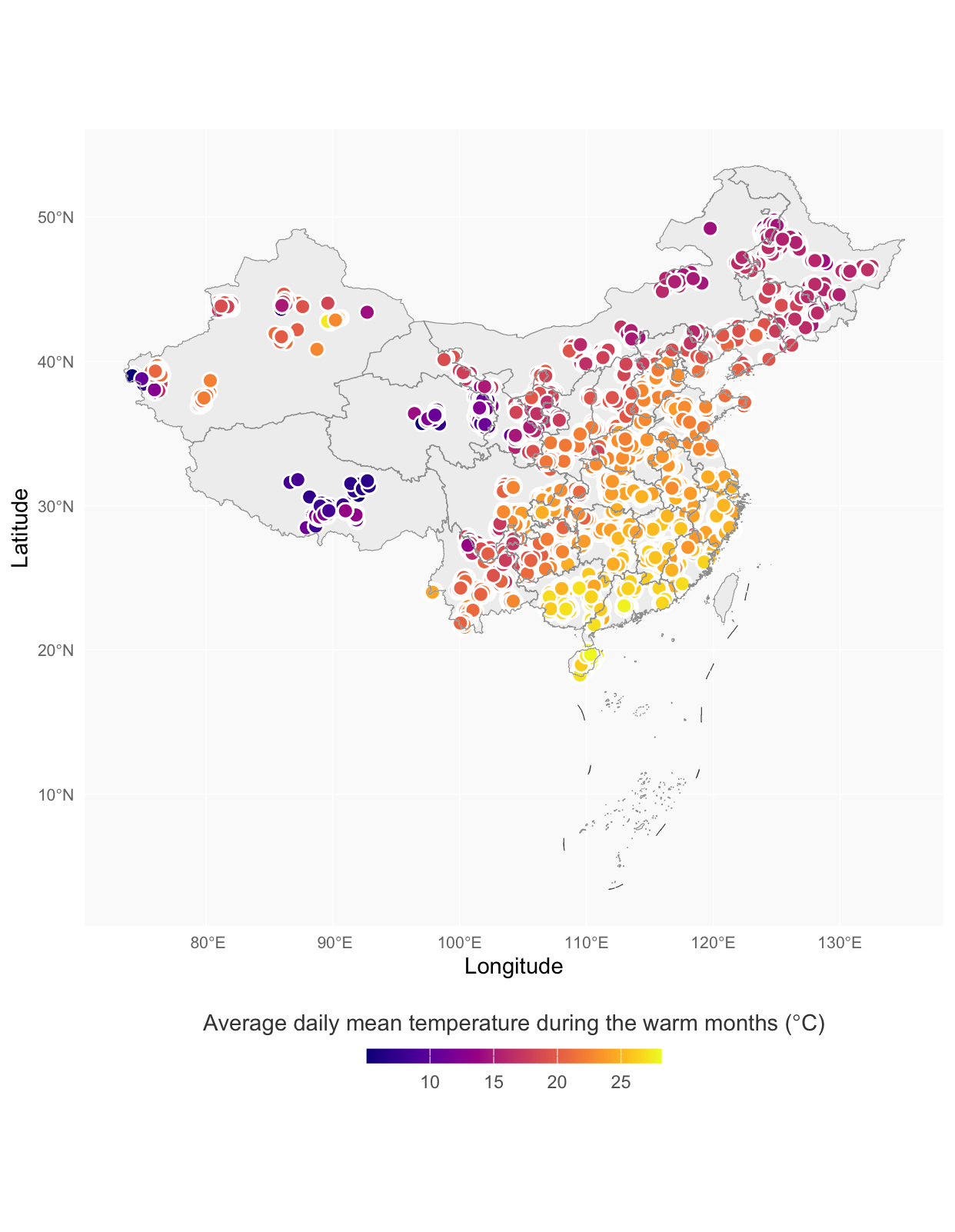


**Figure S1. Spatial distribution of average daily mean temperature during warm months across study locations in China.** This map illustrates the spatial distribution of average daily mean temperatures during warm months (May to October) across all study locations in China from 2009 to 2020. Points represent the geographic locations of under-5 mortality cases.


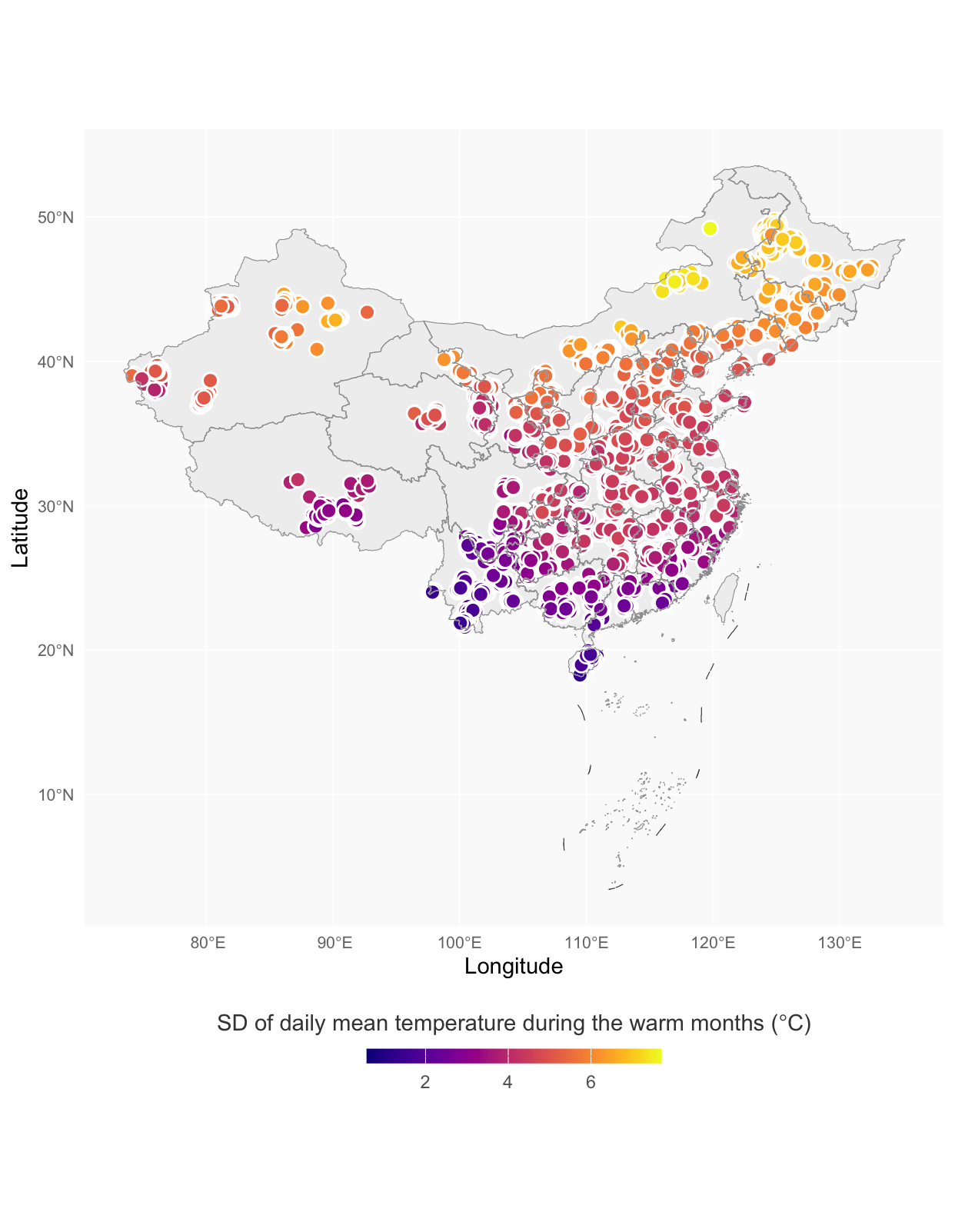


**Figure S2. Spatial distribution of daily mean temperature variability during warm months across study locations in China.** This map illustrates the spatial distribution of temperature variability, represented by the standard deviation (SD) of daily mean temperatures during warm months (May to October) across all study locations in China from 2009 to 2020. Points represent the geographic locations of under-5 mortality cases.


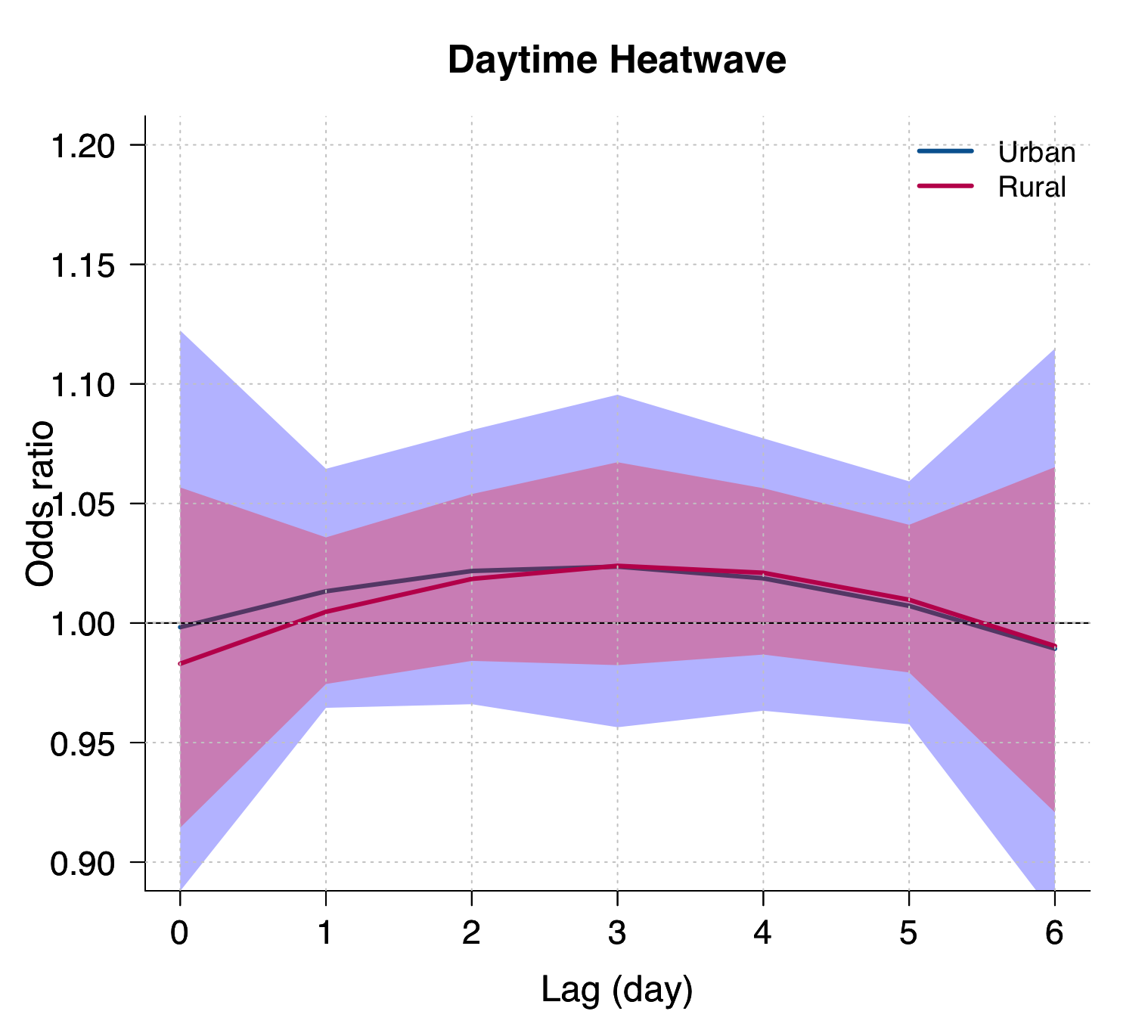


**Figure S3. Lag pattern of daytime heat wave effects on under-5 mortality risk in urban and rural areas.** This figure illustrates the lag effects of compound heat waves on under-5 mortality risk in urban (blue) and rural (red) areas. The y-axis represents odds ratios, while the x-axis shows lag days from 0 to 6. Shaded areas indicate 95% confidence intervals. Daytime heat waves were defined as days when the daily maximum temperature exceeded the local 90th percentile for two or more consecutive days.


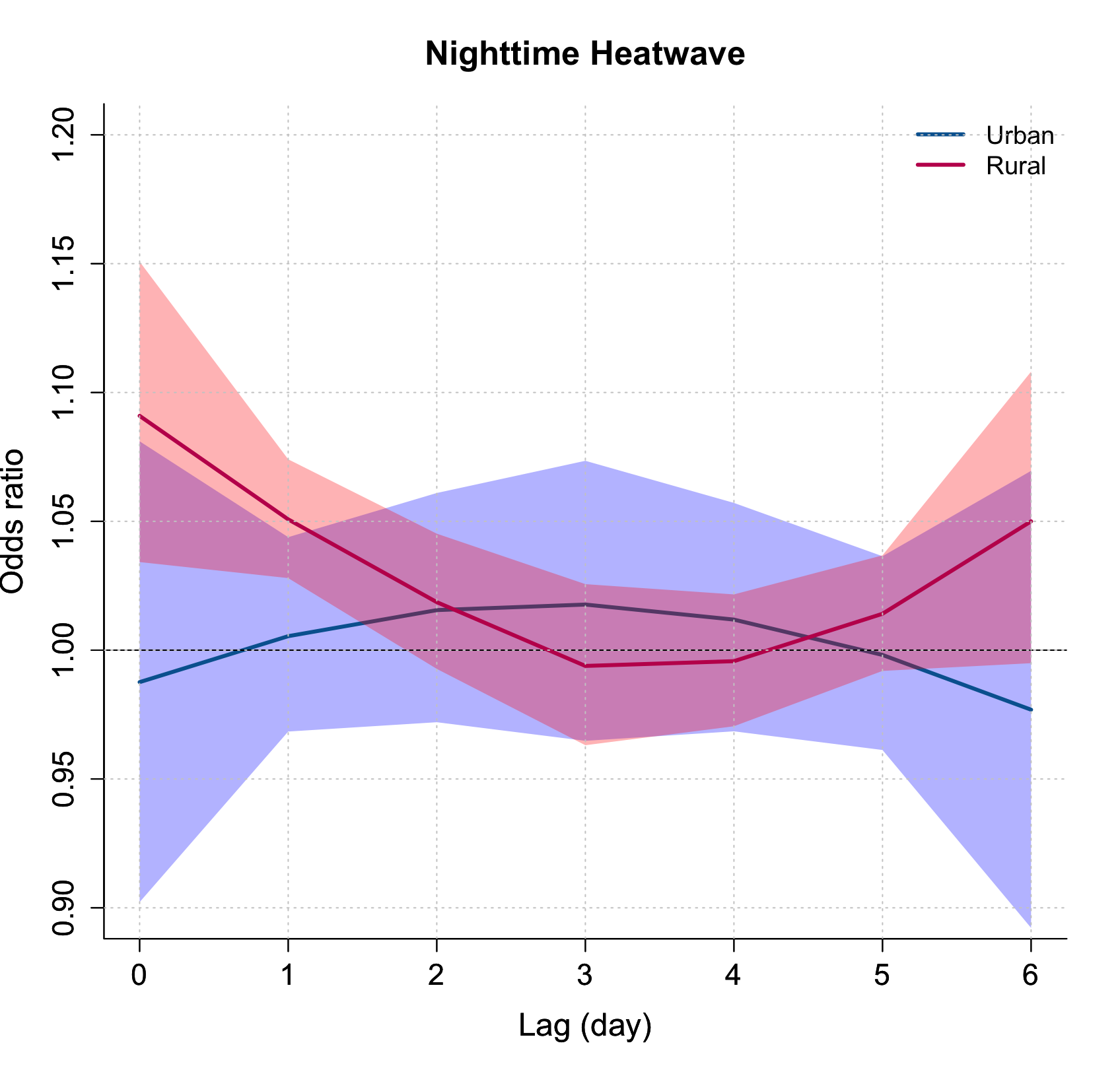


**Figure S4. Lag pattern of nighttime heat wave effects on under-5 mortality risk in urban and rural areas**. This figure illustrates the lag effects of compound heat waves on under-5 mortality risk in urban (blue) and rural (red) areas. The y-axis represents odds ratios, while the x-axis shows lag days from 0 to 6. Shaded areas indicate 95% confidence intervals. Daytime heat waves were defined as days when the daily minimum temperature exceeded the local 90th percentile for two or more consecutive days.

**
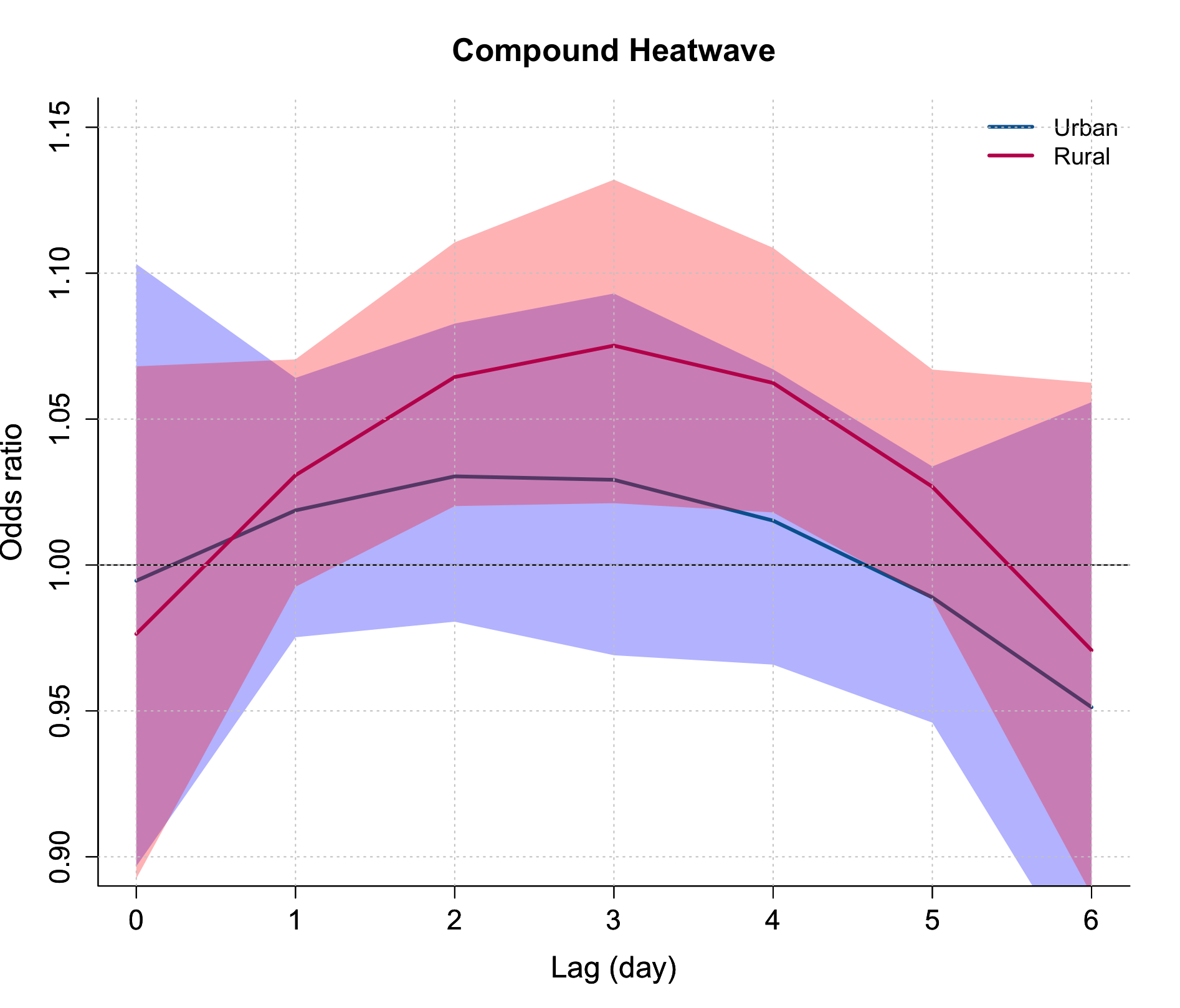
**

**Figure S5. Lag pattern of compound heat wave effects on under-5 mortality risk in urban and rural areas.** This figure illustrates the lag effects of compound heat waves on under-5 mortality risk in urban (blue) and rural (red) areas. The y-axis represents odds ratios, while the x-axis shows lag days from 0 to 6. Shaded areas indicate 95% confidence intervals. Compound heat waves were defined as days when both the daily maximum and minimum temperatures exceeded their respective local 90th percentiles for two or more consecutive days.


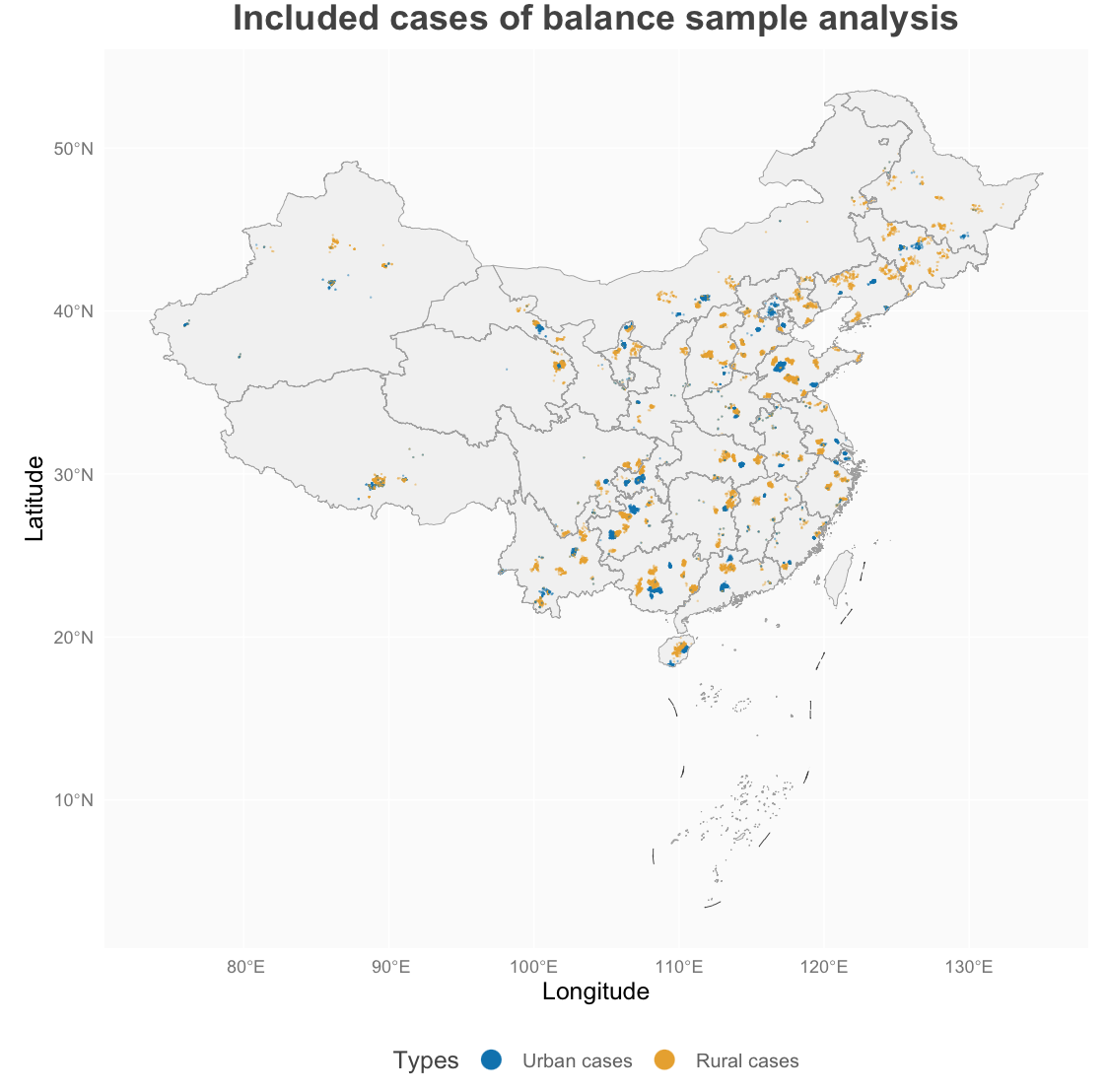


**Figure S6 Spatial distribution of included cases in the balanced sample analysis.** The geographical distribution of matched urban (blue, n=11,264) and rural (red, n=11,264) under-5 mortality cases identified by our balanced sample analysis. Each urban case was matched with its closest rural counterpart having the identical cause of death, with each rural case used only once in the matching process. This one-to-one matching approach ensured equal sample sizes between urban and rural areas while maintaining cause-specific comparability.

**References**

1. Chen R, Cai J, Meng X, et al. Ozone and daily mortality rate in 21 cities of East Asia: how does season modify the association? *Am J Epidemiol* 2014; 180(7): 729-36.

2. Gasparrini A, Guo Y, Hashizume M, et al. Mortality risk attributable to high and low ambient temperature: a multicountry observational study. *The lancet* 2015; 386(9991): 369-75.
